# Supplementary material for: Real-time ultrasound evaluation of CORE muscle activity in a simultaneous contraction in subjects with non-specific low back pain and without low-back pain. Protocol of an observational case-control study
Source: PLoS One. 2023 Aug 10;18(8):e0285441. doi: 10.1371/journal.pone.0285441 (PMC10414640; doi:10.1371/journal.pone.0285441)
Supplement: S1 Appendix — (DOCX) [file pone.0285441.s001.docx]

# **Supporting information**

**Appendix 1. Patient information sheet (hip)**

**Patient information sheet**

**Title of the study**: Real-time ultrasound evaluation of CORE muscle activity in a simultaneous contraction in subjects with non-specific low back pain and without low-back pain. Protocol of an observational case-control study.

Principal Investigators: María Cervera Cano

Sub-investigators: M^a^ del Carmen Sáez García, David Valcárcel-Linares, Samuel Fernández-Carnero, Luis López-González, Tomás Gallego-Izquierdo, Daniel Pecos-Martin.

Department: Department of Physiotherapy

University: University of Alcalá.

City/Country: Madrid (Alcalá de Henares), Spain.

The purpose of this sheet is to inform about the research study to be carried out. You should read this document calmly, and you can take it home with you to think about your possible participation in this study. You can ask the research team all the questions you need to ask (section ‘contact’ at the end of the sheet), and they will answer them as soon as they read them. The participation in this study is completely voluntary and you may withdraw from it at any time without suffering any prejudice.

**Justification of the study and who carries it out**

The study will be conducted at the University of Alcalá in the physiotherapy department by a research team composed of a principal investigators (María Cervera Cano and the secondary investigators ( M^a^ del Carmen Sáez García, David Valcárcel-Linares, Samuel Fernández-Carnero, Luis López-González, Tomás Gallego-Izquierdo, Daniel Pecos-Martin)

To understand why this study will be carried out, it is important to know a few things about non-specific low-back pain.

Low-back pain is defined as pain located between the last rib and the buttock, it can be on one side of the body, the other side or both. The onset of the pain can be insidious, and it is not associated with a specific trauma, infection, tumor or vertebral pathology, which is why it is called 'non-specific' low back pain. Only 10% are classified as specific low back pain.

What it’s known is that low back pain has become one of the biggest public health problems due to its frequency (between 60-85% of the population will suffer from it at some point) and recurrence, which leads to very high costs.

Until now, numerous diagnostic tests have been used to explain low back pain, such as magnetic resonance imaging, computerized tomography (CT), electromyography or X-rays. Despite all the studies carried out using these diagnostic tests, the cause of this pain is still unknown. For this reason, further research is needed

In this study, ultrasound will be used as a novel technique in physiotherapy, to observe the behavior of the muscles that stabilize the trunk in subjects without pain and with pain. This ultrasound examination could allow us to know what the muscular behavior is, in order to answer our research question: Are there any differences between the muscular behavior as a whole in subjects with low back pain?

Due to the lack of information about the origin of this pain, there is some uncertainty regarding treatment in physiotherapy. Therefore, knowing the muscular mechanisms of this pathology could provide new lines of treatment.

**Objectives of the study**

The aim of this study is to observe whether there are differences in the functioning of the muscles that stabilize the trunk between subjects who suffer non-specific low-back pain and those who do not. These differences may be a delay or alteration in the contraction of a muscle group, either because it is weaker or because another muscle group is functioning excessively. That is why evaluating the onset in which the muscles are activated is the main objective of this study. It is thought that subjects with pain could show differences in the contraction time of each muscle and simultaneously.

**Methodology**

This is an observational research, that is to say, the research team will observe the behavior of the muscles through ultrasound imaging at a given moment, which is why it is also called a 'cross-sectional study'. No therapy or intervention will be applied.

If the participant wishes to take part in the study as a case (pain) or as a control (without pain), he or she must contact the research team to make an appointment for the day of measurement. Once enrolled in the study, the participant will go to the Faculty of Physiotherapy at the University of Alcalá on the aforementioned day, where he or she will be provided with all the necessary documents to be completed.

It is important to go to your appointment with a full bladder, as it will help you visualize certain muscle groups better!

Once the necessary documents have been completed and this document has been read, the measurements will begin.

A customized belt that will be place and adapted to each participant. This customized belt will allow a free movement in order to perform the tests. There will be four tests. Each test will be carried out three times in standing and sitting, with rest times in between to avoid tiredness or pain.

Before starting the measurement, the participant must choose an opaque envelope that will randomly assign the order of the test.

The duration of the data collection will be approximately 60 minutes per person. Once the tests have been completed the belt will be removed, and the participant may leave the room. The research team will then save the data obtained for subsequent analysis.

**Potential benefits of the study for the subject**

The study participants will be able to learn, know and observe, through ultrasound, how their muscles are activated during movement. Furthermore, in the case of non-specific low-back pain, the subjects could benefit in the future from the results obtained.

Ultimately, participation in this study could contribute to broadening the knowledge of the scientific community and, therefore, benefit society with the possible new results obtained.

**Risks and/or inconvenience of participating in the study**

As this is an observational case-control study, the risks and/or discomfort that may arise are unlikely or non-existent. No invasive technique or treatment will be carried out that could lead to any new symptomatology.

In the event of experiencing any adverse reaction, the research team must be informed.

**Protection of participant’s rights**

The personal data collected in the study of which the participant has been previously informed, will be processed by the researchers of the University of Alcalá (UAH) in accordance with the Organic Law 3/2018, of Protection of Personal Data and guarantee of digital rights and the General Regulation of Data Protection (EU) 2016/679, with the purpose of processing your participation.

**Data to be collected and processed**

Personal data such as identification data, subject characteristics, and questions of interest to the study will be collected by the *'participant data collection form'*.

The data corresponding to the ultrasound images will be collected through the customized belt that contains the probes. These images and data will be stored in a computer, and later, the researchers will analyze them using specific programs for this purpose.

Other interesting data such as pain (EVA scale) and disability (Oswestry questionnaire) will be collected through self-administered questionnaires, in Spanish and previously validated by the scientific community.

**Data protection and confidentiality**

The data will be processed with the subject consent and within the framework of the educational and research function legally attributed to the University. These data will not be transferred except on request and in the cases provided for by law and will be kept for the time legally established and necessary to fulfil the aforementioned purpose.

The body responsible for the processing data is the general secretary of the university, before whom the rights of access, rectification, suppression, opposition, limitation of processing and portability may be exercised by writing to the Data Protection Officer (Colegio de San Ildefonso, Plaza de San Diego, s/n. 28801 Alcalá de Henares. Madrid) or by e-mail (protecciondedatos@uah.es), attaching a copy of the ID card or equivalent. In case of conflict, an appeal may be lodged with the Spanish Data Protection Agency. For more detailed information, please consult the University's Privacy Policy".

All the results obtained will be recorded in a database which will be kept permanently anonymous by means of encryption. At any time, the participant can leave the study and revoke his or her wish to take part in it. The data we collect as part of this study will be shared with other researchers and students, not your name or images unless we have your explicit consent to do so.

**Contact**

In case of doubt or need, you can contact the principal investigators of the study through the e-mail and phone number indicated below:

Name: María Cervera Cano

Telephone: +34 628 60 64 24

E-mail: [fisioterapiacervera@gmail.com](mailto:fisioterapiacervera@gmail.com)
