# Supplementary material for: Real-time ultrasound evaluation of CORE muscle activity in a simultaneous contraction in subjects with non-specific low back pain and without low-back pain. Protocol of an observational case-control study
Source: PLoS One. 2023 Aug 10;18(8):e0285441. doi: 10.1371/journal.pone.0285441 (PMC10414640; doi:10.1371/journal.pone.0285441)
Supplement: S2 Appendix — (DOCX) [file pone.0285441.s002.docx]

**Appendix 2. Declaration of informed consent.**

# **Declaration of Informed Consent**

I.......................................................................with DNI/NIE ................................. have read the information sheet and have had enough time to consider my decision to participate in the study: *'Real-time ultrasound evaluation of CORE muscle activity in simultaneous contraction in subjects with non-specific low back pain and without low back pain. Observational case-control study'*.

- I affirm that I have been given the opportunity to ask questions and that all have been answered satisfactorily.

- I understand that my participation is voluntary.

- I understand that I may withdraw from the study

o Whenever I wish.

o Without having to explain myself.

o Without personal repercussions.

I freely agree to participate in the study and consent to the access and use of my data under the conditions detailed in the information sheet.

I have received a copy of this document

Participant's signature: Signature of the research team:

Date Date

# **Withdraw consent**

I,......................................................................with DNI/NIE..............................revoke the consent given on (date) ..................... and do not wish to continue participating in the study 'Real-time ultrasound evaluation of CORE muscle activity in simultaneous contraction in subjects with non-specific low back pain and without low back pain. Protocol of an observational case-control study'.

Participant's signature: Signature of the research team:

Date Date

# **Declaration of Informed Consent**

# **(Participant’s copy)**

I.......................................................................with DNI ................................. have read the information sheet and have had enough time to consider my decision to participate in the study: *'Real-time ultrasound evaluation of CORE muscle activity in simultaneous contraction in subjects with non-specific low back pain and without low back pain. Observational case-control study'*.

- I affirm that I have been given the opportunity to ask questions and that all have been answered satisfactorily.

- I understand that my participation is voluntary.

- I understand that I may withdraw from the study

o Whenever I wish.

o Without having to explain myself.

o Without personal repercussions.

I freely agree to participate in the study and consent to the access and use of my data under the conditions detailed in the information sheet.

I have received a copy of this document

Participant's signature: Signature of the research team:

Date Date

# **Withdraw consent**

I,......................................................................with DNI/NIE..............................revoke the consent given on (date) ..................... and do not wish to continue participating in the study 'Real-time ultrasound evaluation of CORE muscle activity in simultaneous contraction in subjects with non-specific low back pain and without low back pain. Protocol of an observational case-control study'.

Participant's signature: Signature of the research team:

Date Date
