# Supplementary material for: Real-time ultrasound evaluation of CORE muscle activity in a simultaneous contraction in subjects with non-specific low back pain and without low-back pain. Protocol of an observational case-control study
Source: PLoS One. 2023 Aug 10;18(8):e0285441. doi: 10.1371/journal.pone.0285441 (PMC10414640; doi:10.1371/journal.pone.0285441)
Supplement: S3 Appendix — (DOCX) [file pone.0285441.s003.docx]

**Appendix 3. Participant data collection form.**

# **Participant Data Collection Form**

Date: ......./......./.........

**Subject code** (to be filled in by the research team): ..................................

## **Identification data:**

Last Name:

First Name:

Age:

NIF:

Telephone number:

E-mail:

Job:

## **Subject's characteristics:**

Gender: Female/Male

- Only for females:
  - Number of pregnancies:
  - Number of births:

Are you pregnant or do you think you might be? YES/NO

Personal medical history:..........................................................................

Height:...........cm. Weight:.......... kg
