# Supplementary material for: Real-time ultrasound evaluation of CORE muscle activity in a simultaneous contraction in subjects with non-specific low back pain and without low-back pain. Protocol of an observational case-control study
Source: PLoS One. 2023 Aug 10;18(8):e0285441. doi: 10.1371/journal.pone.0285441 (PMC10414640; doi:10.1371/journal.pone.0285441)
Supplement: S4 Appendix — (DOCX) [file pone.0285441.s004.docx]

**Appendix 4. Low Back Pain Questionnaire**

# **Low Back pain Questionnaire**

This questionnaire designed by the research team consists of 9 questions that prove to be very important to know if you are a subject suffering from low back pain or if you are asymptomatic. All the answers are protected under the data protection law that you will find at the end of the form.

1. Do you suffer *low back pain* today? YES/NO

If yes, please specify when the low back pain started:……… *(DD/MMM/YYYY)*

1. Have you had *low back*  *pain* in the last year? YES/NO

If yes, have you suffered low back pain for more than three months in the last year? YES/NO

Duration:............................... months/years.

Number of episodes:....................................

1. In case of suffering low back pain, have you been diagnosed with any *spinal disorders* (hernia, scoliosis, spondylosis, etc.) that has been directly related to your lumbar pain? YES/NO

Please, specify: ……………………

1. Have you ever had *sciatica* (pain that radiates electrically in the legs beyond the knee)? YES/NO
2. Have you ever had a *surgery*? YES/NO

If yes, please specify which:....................................................................................

1. Have you had/have you had any kind of *oncological process*? YES/NO

If yes, please specify when the cancer process started:.………….*(DD/MMM/YYYY)*

If yes, is the cancer process ongoing? YES/NO

If yes, please specify when the cancer process ended:.……………..*(DD/MMM/YYYY)*

1. Have you suffered or do you suffer any *depressive disorder*? YES/NO

If yes, please indicate when the depressive disorder started:............... *(DD/MMM/YYYY)*

If yes, please indicate when the depressive disorder ended:............... *(DD/MMM/YYYY)*

1. Do you take any *medication* on a regular basis? YES/NO

If yes, please specify the trade name: ...............................................................................................

If yes, please indicate when the medication was started:............... *(DD/MMM/YYYY)*

If yes, please indicate when the medication was ended:............... *(DD/MMM/YYYY)*

1. Do you take *anti-inflammatory* medication? YES/NO

If yes, please specify the trade name: ........................................

If yes, please indicate frequency: daily/montly/on demand.

1. Do you currently participate in any core exercise program?

If yes, specify frequency:

If yes, how long have you been practicing and how often?...............

If you do not currently participate in any core exercise program but have participated, when was the last time?............................ *(DD/MMM/YYYY)*

Participant sign: Research team sign:

Date: Date:

(*) In accordance with the Organic Law 3/2018, of December 5, on Personal Data Protection and guarantee of digital rights, it is ensured that the data collected by the research team have the commitment of confidentiality, with the legally established security measures, and under no circumstances are transferred or treated by third parties, natural or legal persons, without the prior consent of the client, guardian or legal representative, except in those cases in which it is essential for the proper provision of the service.
