# Supplementary material for: Real-time ultrasound evaluation of CORE muscle activity in a simultaneous contraction in subjects with non-specific low back pain and without low-back pain. Protocol of an observational case-control study
Source: PLoS One. 2023 Aug 10;18(8):e0285441. doi: 10.1371/journal.pone.0285441 (PMC10414640; doi:10.1371/journal.pone.0285441)
Supplement: S1 File — (PDF) [file pone.0285441.s005.pdf]

(A rellenar por la Secretaría)
Cód. CEI: CEIM/HU/2019/41

UNIVERSIDAD DE ALCALÁ, PATRIMONIO DE LA HUMANIDAD

COMITÉ DE ÉTICA DE LA INVESTIGACIÓN Y DE EXPERIMENTACIÓN ANIMAL DE LA UNIVERSIDAD DE ALCALÁ

INFORME

El Comité de Ética de la Investigación y de Experimentación Animal de la Universidad de Alcalá 15 de julio de 2019 ha evaluado el proyecto del trabajo de fin de master titulado ***“Diferencias en la actividad electromiográfica y en grosor en una situación de activación simultánea de la musculatura del CORE. Estudio observacional de casos y controles”***, presentado por Dª. María Cervera Cano y Dª. Carmen Sáez García estudiantes del Master de Fisioterapia de esta Universidad.

Analizados los extremos acreditados en el expediente, el Comité considera que el proyecto del trabajo de fin de master y el procedimiento evaluado son correctos desde el punto de vista ético y metodológico, y por lo tanto da su informe FAVORABLE.

Y para que conste, se firma este informe en Alcalá de Henares, a 22 de noviembre de 2019.

F. Javier de la Mata de la Mata
Presidente del CEI y EA

Código Seguro De Verificación:	Nii7gU7xSaftIJ10anmCJg==	Estado	Fecha y hora
Firmado Por	Francisco Javier De La Mata De La Mata - Vicerrector de Investigación Y Transferencia	Firmado	25/11/2019 11:15:46
Observaciones		Página	1/1
Url De Verificación	https://vfirma.uah.es/vfirma/code/Nii7gU7xSaftIJ10anmCJg==		
