# Supplementary material for: Real-time ultrasound evaluation of CORE muscle activity in a simultaneous contraction in subjects with non-specific low back pain and without low-back pain. Protocol of an observational case-control study
Source: PLoS One. 2023 Aug 10;18(8):e0285441. doi: 10.1371/journal.pone.0285441 (PMC10414640; doi:10.1371/journal.pone.0285441)
Supplement: Attachment — Submitted filename: Response to reviwers2.pdf [file pone.0285441.s006.pdf]

Dr. Samuel Fernández Carnero
Department of Nursing and Physiotherapy
University of Acala, Faculty of Medicine and Health Sciences.
Pza. San Diego, s/n 28801 - Alcalá de Henares (Madrid)
España
+34 91 885 50 00

Editorial Office

PlosOne

20th December 2022

Dear Academic Editor

Please find enclosed one complete copy of our original investigation, titled: "**Real-time ultrasound evaluation of CORE muscle activity in a simultaneous activation in subjects with non-specific low back pain and without low-back pain. Protocol of an observational cross-sectional study**" which we would like to be considered for publication in "**PlosOne**" as an original article.

We have reviewed all the recommendations received by the reviewers and detailed the changes in this cover letter. The responses for the reviewer are written in red. The main document has been edited with the track changes active to be reviewed easily.

The contents of the manuscript have not been copyrighted or published previously.

This paper has not been submitted simultaneously to another journal, in whole or in part, it reports previously unpublished work, and if accepted it will not be published in the same form, in any language, without the consent of the publishers.

With our appreciation in advance, we look forward to your decision on this article in due time. Please do not hesitate to contact me meanwhile if there are any questions regarding our manuscript.

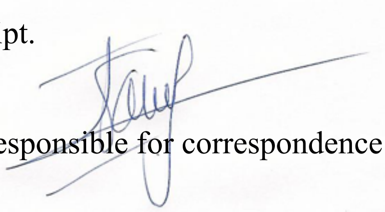

The author responsible for correspondence is:

Dr. Samuel Fernández Carnero, PhD, MsC, PT

University of Acala, Faculty of Medicine and Health Sciences.

Telephone number: +34 620895315.

E-mail address: samuelfernandezcarnero@gmail.com

Campus Científico-Tecnológico, Crta. de Madrid-Barcelona, Km. 33,600, 28805 Alcalá de Henares, Madrid (Spain).

Response to Reviewers

Journal Requirements:

When submitting your revision, we need you to address these additional requirements.

1. Please ensure that your manuscript meets PLOS ONE's style requirements, including those for file naming. The PLOS ONE style templates can be found at https://journals.plos.org/plosone/s/file?id=wjVg/PLOSOne_formatting_sample_main_body.pdf and https://journals.plos.org/plosone/s/file?id=ba62/PLOSOne_formatting_sample_title_authors_affiliations.pdf

Updated. Thank you

2. We note that your submission may include questionnaire items that may have been previously published. The reproduction of previously published work has implications for the copyright that may apply to these publications. We would be grateful if you could clarify whether you have obtained permission from the original copyright holder to republish these items under a CC BY license. If you have not obtained permission to publish these items please remove them from your manuscript. You may wish to replace the text you have removed with relevant question numbers/ brief descriptions of each item; please be sure to include any relevant references and in-text citations.

The participants have been included according to the definition of chronic low back pain by different authors. These references have been introduced in each of the questions posed in the questionnaire designed by the research team. Thank you very much for your contribution.

3. Thank you for stating the following financial disclosure:
"The funders had and will not have a role in study design, data collection and analysis, decision to publish, or preparation of the manuscript."

At this time, please address the following queries:

a) Please clarify the sources of funding (financial or material support) for your study. List the grants or organizations that supported your study, including funding received from your institution.

b) State what role the funders took in the study. If the funders had no role in your study, please state: "The funders had no role in study design, data collection and analysis, decision to publish, or preparation of the manuscript."
c) If any authors received a salary from any of your funders, please state which authors and which funders.
d) If you did not receive any funding for this study, please state: "The authors received no specific funding for this work."

Please include your amended statements within your cover letter; we will change the online submission form on your behalf.

Line 420. "The authors received no specific funding for this work." Thank you.

4. Thank you for stating the following in your Competing Interests section:
"The authors have declared that no competing interests exist."

Please complete your Competing Interests on the online submission form to state any Competing Interests. If you have no competing interests, please state "The authors have declared that no competing interests exist.", as detailed online in our guide for authors at http://journals.plos.org/plosone/s/submit-now This information should be included in your cover letter; we will change the online submission form on your behalf.

Line 421: "The authors have declared that no competing interests exist."

5. We note that you have stated that you will provide repository information for your data at acceptance. Should your manuscript be accepted for publication, we will hold it until you provide the relevant accession numbers or DOIs necessary to access your data. If you wish to make changes to your Data Availability statement, please describe these changes in your cover letter and we will update your Data Availability statement to reflect the information you provide.

6. Please upload a copy of Figure 3, to which you refer in your text on page 7. If the figure is no longer to be included as part of the submission, please remove all reference to it within the text.

[Note: HTML markup is below. Please do not edit.]

Reviewers' comments:

Reviewer's Responses to Questions

Comments to the Author

1. Does the manuscript provide a valid rationale for the proposed study, with clearly identified and justified research questions?

The research question outlined is expected to address a valid academic problem or topic and contribute to the base of knowledge in the field.

Reviewer #1: Yes

---

2. Is the protocol technically sound and planned in a manner that will lead to a meaningful outcome and allow testing the stated hypotheses?

The manuscript should describe the methods in sufficient detail to prevent undisclosed flexibility in the experimental procedure or analysis pipeline, including sufficient outcome-neutral conditions (e.g. necessary controls, absence of floor or ceiling effects) to test the proposed hypotheses and a statistical power analysis where applicable. As there may be aspects of the methodology and analysis which can only be refined once the work is undertaken, authors should outline potential assumptions and explicitly describe what aspects of the proposed analyses, if any, are exploratory.

Reviewer #1: Yes

---

3. Is the methodology feasible and described in sufficient detail to allow the work to be replicable?

Descriptions of methods and materials in the protocol should be reported in sufficient detail for another researcher to reproduce all experiments and analyses. The protocol should describe the appropriate controls, sample size calculations, and replication needed to ensure that the data are robust and reproducible.

Reviewer #1: Yes

---

4. Have the authors described where all data underlying the findings will be made available when the study is complete?

The PLOS Data policy requires authors to make all data underlying the findings described in their

manuscript fully available without restriction, with rare exception, at the time of publication. The data should be provided as part of the manuscript or its supporting information, or deposited to a public repository. For example, in addition to summary statistics, the data points behind means, medians and variance measures should be available. If there are restrictions on publicly sharing data—e.g. participant privacy or use of data from a third party—those must be specified.

Reviewer #1: No

---

5. Is the manuscript presented in an intelligible fashion and written in standard English?

PLOS ONE does not copyedit accepted manuscripts, so the language in submitted articles must be clear, correct, and unambiguous. Any typographical or grammatical errors should be corrected at revision, so please note any specific errors here.

Reviewer #1: Yes

---

6. Review Comments to the Author

Please use the space provided to explain your answers to the questions above and, if applicable, provide comments about issues authors must address before this protocol can be accepted for publication. You may also include additional comments for the author, including concerns about research or publication ethics.

You may also provide optional suggestions and comments to authors that they might find helpful in planning their study.

(Please upload your review as an attachment if it exceeds 20,000 characters)

Reviewer #1: This is a study protocol that investigates the activation time of core muscles via ultrasound in subjects with and without low back pain.

Abstract:

“Observational case-control study." Why did the authors mention “Protocol of an observational cross sectional study” in the title?

Line 4. Reviewed and changed. Apologies, it was a mistake. Thank you.

Method:

Who will diagnose NSLBP? Physician or questionnaire?

Lines 131 and 754.

A questionnaire will be given to the participant. This questionnaire is designed by the research team and contains information regarding to the inclusion and the exclusion criteria described in the scientific literature. An unblinded subinvestigator (D.V.L and L.L.G) will archive the questionnaires so as not to break the blind.

Thank you

Will the authors consider acute NSLBP or chronic NSLBP? “History of LBP at least three full weeks” for example, a person with 25 days of NSLBP is considered to have acute LBP, while “more than three months in the last year” is a definition for chronic NSLBP.

Line 144. Only participants with chronic nonspecific low back pain (CNSLBP) defined as twelve weeks last year.

In Oliveira's clinical practice guideline (reference 2), it does provide information on pain duration and classification into acute, subacute and chronic, so this inclusion criterion and the definition of this protocol have been changed according to:

> Three guidelines [1, 11, 28] defined acute LBP as less than 4 weeks duration, two guidelines [6, 26] specified less than 6 weeks duration and four guidelines [15, 25, 30, 31] defined acute LBP as less than 12 weeks duration. The Canadian guideline [7] defined acute and subacute LBP as less than 12 weeks duration but without specifying the cutoffs for each one. All guidelines defined chronic LBP as more than 12 weeks' duration. *(Oliveira CB, Maher CG, Pinto RZ, Traeger AC, Lin CWC, Chenot JF, et al. Clinical practice guidelines for the management of non-specific low back pain in primary care: an updated overview. Eur Spine J. noviembre de 2018;27(11):2791-803.*

The article: *Moissenet F, Rose-Dulcina K, Armand S, Genevay S. A systematic review of movement and muscular activity biomarkers to discriminate non-specific chronic low back pain patients from an asymptomatic population. Sci Rep. diciembre de 2021;11(1):5850.* uses this criterion to select the sample for his study, but it is not a definition of the duration of low back pain.

Thank you so much for the correction.

Will the authors include patients with a history of recent participation in core exercises in the past six months?

Line 829. We include patients who have participated in core exercises or have practice sports in the last six months as we did not consider it. Upon reading your question, we have updated the questionnaire with a question about sport and frequency. In this way we will try to draw conclusions. Thank you very much.

"Balance Mass Index (BMI)"? body or balance?

Line 379. Body Mass index.

Apologies. Thank you

Did the authors consider the confounding effect of food consumption on the muscle thickness evaluations? (Eur Spine J. 2011 Aug;20(8):1312-7. The effect of food consumption on the thickness of abdominal muscles, employing ultrasound measurements) (Man Ther. 2015 Feb;20(1):194-9. Reversal time of postprandial changes of the thickness of abdominal muscles employing ultrasound measurements).

The confounding effect of food consumption on the muscle thickness evaluations will not be consider. After a critical reading, the article mentioned is an observational study performed only in men, it is shown that different amounts of food are given for such a heterogeneous sample (very large SD) without considering age, comorbidities, metabolism or even BMI, apparently. They measure, moreover, in a relaxed position and do not give results on the amount of muscle contraction. They do not offer results on how much time would be optimal for food consumption not to affect muscle thickness at rest, nor the amount of food it would be advisable to ingest. They further conclude that "To minimize this error, it can be suggested that the time between food intake and ultrasound measurements on different days should be consistent for each person". We think that controlling for this factor is somewhat complicated. The question we would be interested in knowing is whether the contraction-relaxation ratio would be affected by food intake in a short period of time or whether the tension exerted by the stomach affects the functionality of the muscle, even so we insist that the amount of food ingested is key to corroborate these results.

Thank you very much for your contribution as we had not considered this possible confounding factor.

Will the participants be taught how to perform ADIM before measurements?

Line 238. Yes, the participant will be taught to perform ADIM maneouver before performing the measurement (1.Teyhen DS, Miltenberger CE, Deiters HM, Del Toro YM, Pulliam JN, Childs JD, et al. The use of ultrasound imaging of the abdominal drawing-in maneuver in subjects with low back pain. J Orthop Sports Phys Ther. 2005;35(6):346-55.

Where will the authors assess the internal oblique, external oblique, and transverse abdominis?

Line 297. Table 2, first line. LAW (IO, EO, TRA) Distance (cm) between the superficial and deep border of each muscle belly, which is marked by the hyperechoic fascial lines. the fascial lines will not be included in the measurement.

Considering the effect of sex, age, and BMI on muscle thickness, why won't the authors adjust their analysis for sex, age, and BMI? (J Physiol Anthropol. 2016 Aug 23;35(1):17. Measurement of superficial and deep abdominal muscle thickness: an ultrasonography study)

Line 380. We found your contribution very interesting and, after reading the article provided, it fits perfectly with our analysis. Thank you very much.

Is the length of the VAS measurement tool 10 cm? The image in the appendix seems longer than 10 cm.

Yes. It is 10 cm. Thank you

---

7. PLOS authors have the option to publish the peer review history of their article (what does this mean?). If published, this will include your full peer review and any attached files.

If you choose “no”, your identity will remain anonymous but your review may still be made public.

Do you want your identity to be public for this peer review? For information about this choice, including consent withdrawal, please see our Privacy Policy.

Reviewer #1: No
